# Primary female breast sarcoma: clinicopathological features, treatment and prognosis

**DOI:** 10.1038/srep31497

**Published:** 2016-08-11

**Authors:** Ming Yin, Heath B. Mackley, Joseph J. Drabick, Harold A. Harvey

**Affiliations:** 1 Division of Hematology and Oncology, Penn State Hershey Cancer Institute, Hershey, USA; 2 Division of Radiation Oncology, Penn State Hershey Cancer Institute, Hershey, USA

## Abstract

Primary breast sarcoma (PBS) is a rare and heterogeneous group of malignancies with limited publications. We obtained data from the Surveillance, Epidemiology, and End Results Program and performed analysis to determine clinicopathological characteristics of PBS and estimate their associations with overall survival (OS) and cancer-specific survival (CSS). Median age of PBS was 55–59 years and median OS was 108 months. Age, overlap or entire breast involvement, tumor histology, and tumor spread were associated with poor survival outcomes. In the multivariable analysis, tumor size, lymph node involvement, distant metastasis and histologic grade were correlated with survival outcomes (*P* < 0.001). In M0 patients, mastectomy was associated with worse survival outcomes compared with breast conservative surgery (BCS) (adjusted hazard ratio [adjHR], 1.80; 95% CI, 1.31–2.47), regardless of tumor size, tumor grade, tumor histology or radiation history. Adjuvant radiation improved survival outcomes in patients with tumor size >5 cm (adjHR, 0.63; 95% CI, 0.43–0.91), but not in patients with tumor size ≤ 5 cm. Our study demonstrated clinicopathological characteristics of PBS in the US population and supports performing BCS if R0 resection can be achieved, with radiation if tumor size is over 5 cm.

Breast sarcoma, excluding phyllodes tumor, is an extremely rare and heterogeneous group of malignancies, constituting less than 1% of total breast malignancies and less than 5% of all soft tissue sarcomas (STS)^1^. It can be divided into two categories: de novo development (primary) or therapy-related development (secondary). Although clinical features of primary breast sarcoma (PBS) mimic mammary adenocarcinoma in some ways, it conveys a high risk of recurrence and carries a significantly worse prognosis.

Due to its rarity, published literature is limited and confined to small retrospective case reviews and case reports. Optimal care is poorly-defined because information from previous studies is insufficient and inconsistent. In general, breast sarcoma is diagnosed by core or excisional biopsy. Staging is based on American Joint Committee on Cancer (AJCC) 7 for STS, which incorporates histologic grade (G), tumor size (T), node status (N), and distant metastases (M). There is no definitive consensus regarding PBS treatment, and current recommendations are derived from small retrospective case reviews and extrapolated from non-breast STS studies. Complete resection with negative margins (R0) is strongly recommended for curative intent. However, there is a debate of optimal surgical methodologies between breast conservative surgery (BCS) versus mastectomy. The role of radiotherapy and chemotherapy in non-metastatic PBS is also not clear.

The current study utilizes a US population database to analyze a large series of women diagnosed with PBS. The primary objective was to determine clinicopathological characteristics of the PBS patient population and identify patient, pathologic, and treatment characteristics that predict survival outcomes.

## Results

### Patient characteristics and survival outcomes

**Figure 1:**
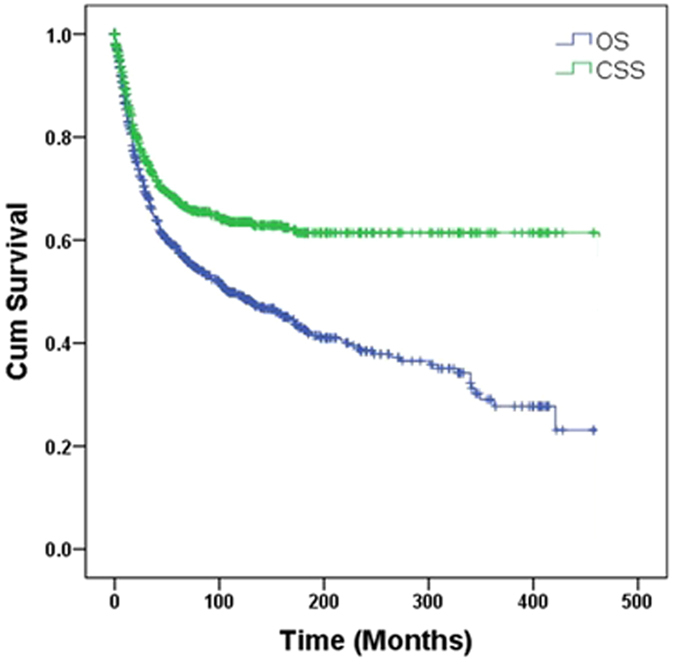
Kaplan-Meier curves for overall survival and cancer-specific survival of the whole PBS patient population.

Figure 1 showed Kaplan-Meier survival curves for OS and CSS of the whole patient population. The OS and CSS curves overlapped for 25 months after diagnosis, reflecting CSS as the predominant cause of mortality in the early phase. CSS curve then plateaued at approximate 100 months, while OS curve continued a downward trend, reflecting a substantially increased non-cancer death risk in the later phase.

Clinical and pathological characteristics of the patients are shown in Table 1. We could roughly divide patients into 25% and 75% percentile by age because the SEER database reports patient age by groups with 5-year interval (e.g. 60–64 years). There were a total of 785 patients with a median age between 55–59 years [range, 15 to over 85 years]. The median OS was 108 months (95% CI, 73.7–142.3) and the main histologic type was angiosarcoma (32.1%). Most PBS involved more than one quandrant (overlap plus entire, 54.3%), and had no metastasis at presentation. 94.7% patients received surgery, while 29.9% patients received radiotherapy, most of which was adjuvant. Only 13 patients received radiotherapy before surgery.

We then examined the associations between OS, CSS and clinicopathologic characteristics to identify confounding factors. Age, more than quandrant involvement, and tumor spread were associated with poor OS and CSS. Caucasians seemed to have an increased cancer-specific death, compared with other ethnicities. Histologically, fibrosarcoma and liposarcoma were associated with better OS and CSS, while osteosarcoma was associated with a worse OS. Patients who received surgery had better survival outcomes. Radiotherapy did not seem to impact OS and CSS in unselected patients.

### Validation of AJCC staging system

The SEER historic stage is not clinically applicable, and the “localized” or “regional” stages are not interchangeable with N0 and N + without metastasis. We re-organized the data by tumor size, node involvement and distant metastasis. The median tumor size was 4.5 cm [range, 0.1 to 48.4 cm]. There were 2.9% positive lymph node and 7.8% distant metastasis at presentation. T, N, M and G status were highly correlated with OS and CSS outcomes (*P* < 0.001) (Table 2 and Supplementary Table 1). Patients with G1 and G2 appeared to have similar OS and CSS outcomes, while patients with Gx seemed to have a similar OS and a relatively better CSS than patients with G3 grade. Our results also showed a significant worse prognosis in patients with G3 or Gx grade, compared with patients with a lower grade of G1 (Table 2 and Supplementary Table 1).

### Impact of treatment modality on survival outcomes

**Figure 2:**
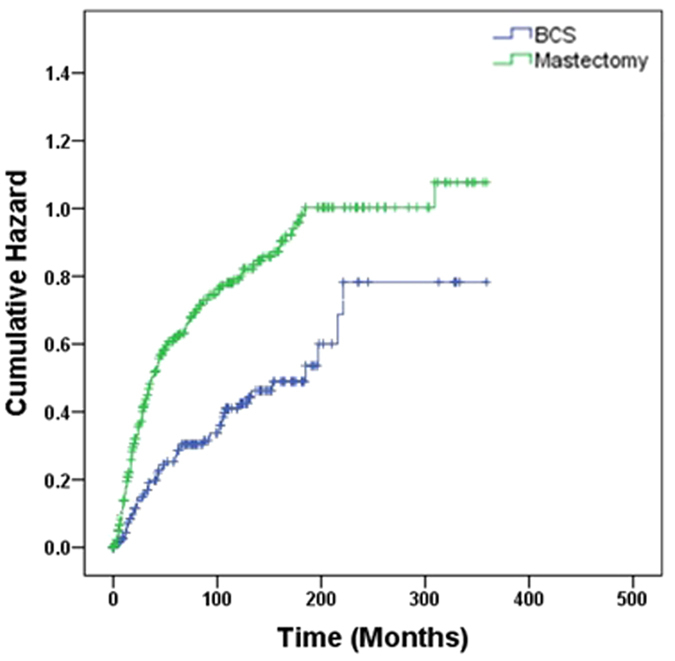
Cumulative probability of death in comparison of breast conservative surgery versus mastectomy in all patients.

As with STS arising in other parts of the body, a multidisciplinary approach has been proposed to treat PBS. We assumed that patients received chemotherapy or best supportive care if neither surgery nor radiation was documented. Compared with mastectomy, BCS was associated with better OS in M0 patients (HR, 1.96; 95% CI 1.48–2.61) (Fig. 2), and in subgroups stratified by tumor size, tumor grade, tumor histology, and radiation history (Table 3).

**Figure 3:**
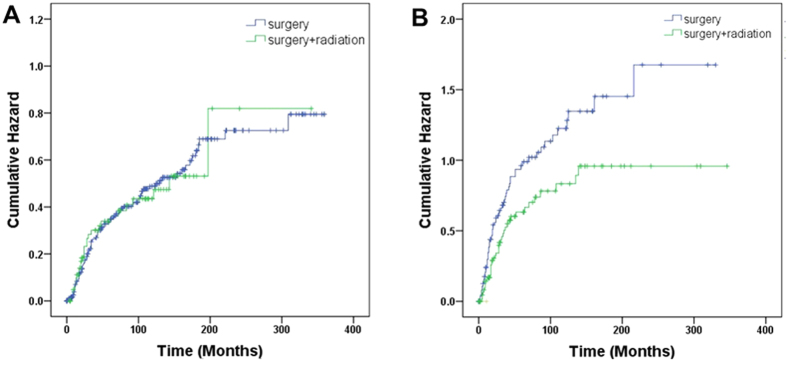
Cumulative probability of death in comparison of surgery alone versus surgery plus radaition. (**A,B**), by ≤5 and >5 cm tumor size.

We then divided patients by tumor size and distant metastasis to compare different treatment modalities. As shown in Fig. 3, surgery plus radiotherapy did not provide OS benefit in T1M0 PBS (HR, 0.96; 95% CI 0.63–1.45; adjHR, 1.12; 95% CI, 0.73–1.71), but was associated with a 36% reduction of death risk in T2M0 PBS (HR, 0.64; 95% CI 0.44–0.92; adjHR, 0.63; 95% CI, 0.43–0.91). In M1 PBS, local treatment by surgery or radiation did not seem to impact overall survival, compared with chemotherapy/best supportive care (data not shown). Similar results were obtained by CSS analyses (Supplementary Table 2).

Since surgical modalities impact outcomes, a chi-squared test was performed and did not detect an imbalance of BCS and mastectomy in surgery versus surgery plus radiation groups (χ^2^ 1.448, *P* = 0.49). Comparison of mastectomy versus mastectomy plus radiation showed a death reduction favoring combination treatment in T2M0 tumor (HR, 0.64; 95% CI 0.44–0.94; adjHR, 0.68; 95% CI, 0.46–1.00).

### Risk stratification of short-term vs. long-term survival (CART analysis)

Prognosis of PBS is influenced by multiple patient, pathological and treatment-related factors beyond TNM staging system. We built a decision tree to screen for parameters that are useful to determine short-term survival (≤4 years) versus long-term survival (>4 years) from all variables that might impact survival outcomes (i.e., age, race, histology, tumor site, tumor size, node status, distant metastasis, tumor grade, surgical modality, and radiation history). We excluded patients with missing information (including Gx) and patients alive with less than 4 years follow-up due to uncertainly of survival status. The final sample size was 434, and the median OS for short-term and long-term survival were 15 and 92 months.

**Figure 4:**
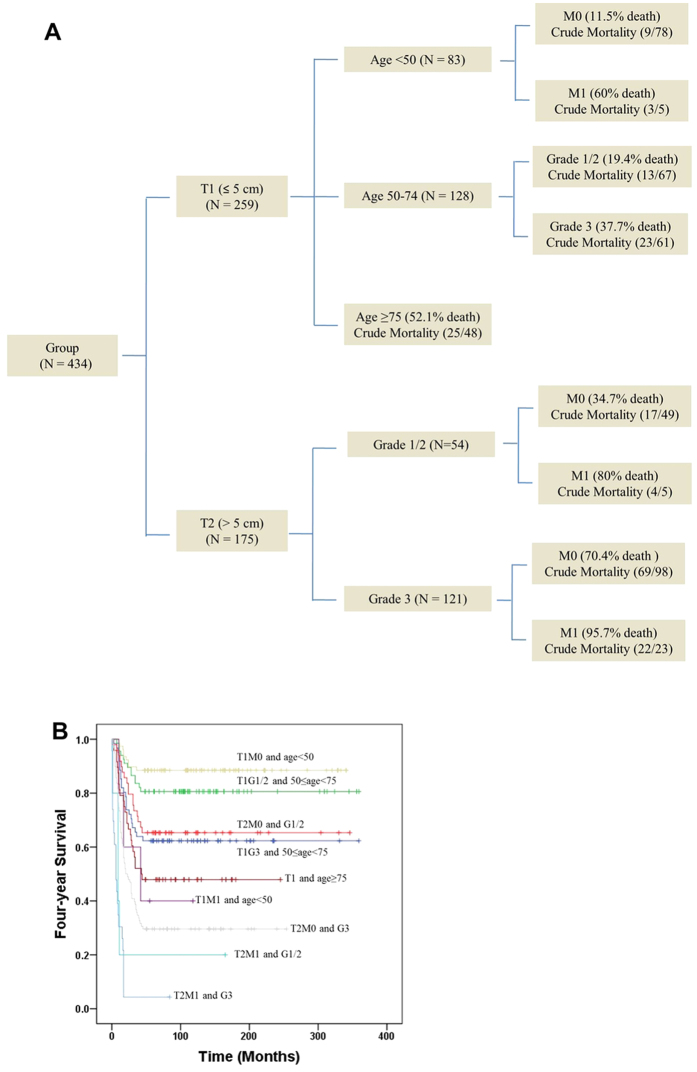
Risk stratification of short-term versus long-term survival. (**A**) Classification and regression tree analysis for stratification of 4-year death risk. (**B**) Kaplan**-**Meier survival curves by selected combination of clinicopathological factors.

The generated tree model (Fig. 4A) properly classified 82.3% short-term survival and 63.2% long-term survival outcomes in 10-fold cross-validation. The first split in the decision tree was tumor size, followed by split of age and tumor grade, respectively. Tumor grade and distant metastasis were the third splitters, while none of the treatment modality was selected by decision tree analysis. A total of nine terminal nodes were identified, with a four-year death risk ranging from 11.5% to 95.7%. The corresponding survival curves (Fig. 4B) confirmed a nice separation of four-year survival outcomes by selected combination of clinicopathological factors.

### Second analysis with imputated datasets

Five imputated datasets were generated, filling missing variables. Our conclusions above were not significantly changed in each impuated dataset or pooled dataset analysis (Supplementary Table 3).

## Discussion

The rarity of PBS precludes any prospective study and poses significant challenges in its diagnosis, treatment and research. The current study of 785 women from SEER database represents the largest reported series to date and allows us to explore this enigmatic disease with relatively sufficient power. Major findings include clinicopathological features of PBS in the US population, validation of AJCC 7 STS staging in PBS, superior survival outcomes associated with BCS compared with mastectomy, and a significant survival benefit of adjuvant radiotherapy in tumor size over 5 cm.

Historically, published studies of PBS were limited by small sample-size, ranging from 25 to 83 patients in individual studies. There is a wide variation in the described clinicopathological characteristics of reported case series, such as the median age of diagnosis (30–60s years old), 5-year survival rate (14–90%), local recurrence rate (15–73%) and common subtype of PBS^2^,^3^,^4^,^5^,^6^,^7^. This inconsistent information precludes the ability to draw firm clinical recommendations. The SEER database is an authoritative source of cancer incidence and survival, covering 28% of the US population. In this large dataset, we revealed the clinicopathological characteristics of the US PBS patient population. PBS typically occurred in women in the fifth decade with a range from the teens to over 85 years. Age was associated with a significantly increased death risk, which was not found in previous studies. Despite the potential of local aggressive growth and distant metastasis, women with PBS had a relatively favorable prognosis with a median OS of 108 months. However, survival time decreased exponentially in the presence of regional and distant tumor spread. Histologically, the leading subtype was angiosarcoma (32.1%), which was previously thought to carry adverse prognosis, compared with other histologies. However, our study demonstrated that osteosarcoma and fibrosarcoma/liposarcoma might have the worst and the best prognosis, respectively. In epithelial breast cancer, medial tumor sites negatively impact survival due to occult internal mammary lymph node involvement^8^. In PBS, tumor sites (quandrant or central) did not appear to impact survival outcomes, probably because PBS rarely spread to lymph nodes (3%). The poor survival outcomes associated with overlap or entire breast involvement seemed to reflect tumor size as an important prognostic factor. Therefore, tumor site was not considered a confounding factor in our analysis.

Accurate staging is of utmost importance in discussing patient outcomes and in determining risk-adaptive treatment. Since 2010, AJCC 7^th^ edition replaced AJCC 6^th^ edition and made several changes, including adoption of the FNCLCC system, omission of tumor depth, re-group of tumor grade and lymph node involvement in staging, etc. However, STS is a significantly heterogeneous group of tumors consisting of different histologies and arising from different anatomic sites. It is known that primary site does have an impact on outcomes^9^, and therefore staging of PBS by general STS may miss some tissue-specific caveats. By validating the performance of current staging system in PBS, our results supported the changes made in AJCC 7 regarding T, N and M, and identified additional risk groups by refining tumor size (≤ 2, 2–5, 5–10 and >10 cm). High tumor grade seemed to be associated with worse survival outcomes (median OS: 340, 221, 42 and 87 months for G1, G2, G3 and Gx). Although Gx was associated with a worse OS than G1/G2, this result should be taken with a grain of salt because we used WHO tumor differentiating score, instead of standard FNCLCC for tumor grading. Most PBS patients in SEER database were diagnosed before 2010 when FNCLCC system was not widely adopted and Gx was recorded but not used in tumor staging.

Current recommendations for PBS treatment are derived from small retrospective case reviews and extrapolated from non-breast STS studies^10^,^11^. Direct evidence from PBS suggests that both BCS and mastectomy seem feasible, if R0 resection can be achieved^3^,^6^. The role of radiotherapy after R0 resection is not clear and there is no consensus criteria for whom should get adjuvant radiotherapy^6^,^12^,^13^. Our study showed that radiotherapy plus surgery provided survival advantage in tumor size over 5 cm, compared with surgery alone. This finding seems to mirror radiotherapy in extremity sarcoma^14^,^15^. Additionally, there appeared some benefit of radiation after mastectomy, which could be due to difficulty to achieve adequate safety margin in large-size, deep-seated PBS. In patients with M1 disease, local treatment by surgery or radiation did not seem to alter the natural course.

Interestingly, BCS was associated with significant better survival outcomes, compared with mastectomy. This finding was consistent with several large studies in epithelial breast cancer and malignant breast phyllodes tumor, which showed superior CSS by BCS^16^,^17^,^18^. Patients who received mastectomy may have large tumor size and high tumor grade, which were intrinsically associated with poor prognosis. However, the survival advantage remains in subgroup analysis, regardless of tumor size, tumor grade, tumor histology or radiation history. The universal survival advantage in different breast malignancies may imply if some unmeasured/unknown variables, which were intrinsically associated with BCS or mastectomy, biased the findings. Adjuvant chemotherapy is unlikely to be the culprit because it is only used in selected high-risk cases in PBS and is rarely used in phyllodes tumor. In epithelial breast cancer, current guidelines do not differentiate between BCS and mastectomy in determining adjuvant therapy. Marren *et al*. suggested that adjuvant radiation, a standard treatment after lumpectomy in epithelial breast cancer, might explain the survival difference by eliminating residual tumor cells^19^. However, our study and the study from phyllodes tumor showed better survival outcomes in BCS groups, regardless of radiotherapy^17^. Some variables, such as performance status and comorbidities, may contribute to survival difference and were not adjusted in our analysis. However, patients receiving mastectomy are not generally weaker or sicker than patients receiving BCS in practice. Other factors such as socioeconomic status and differences in tumor biology (e.g. lymphovascular invasion or extranodal invasion) may contribute to survival differences. We concur with Huang *et al*.^20^ and would not expect a large impact that overrides our findings. Further investigations are required to understand the reasons. In summary, our data provided direct evidence supporting National Comprehensive Cancer Network and European Society of Medical Oncology guidelines in applying treatment principles of extremity sarcoma to PBS regarding wide excision surgery and adjuvant radiation if tumor size is over 5 cm. We further suggest that BCS is still feasible in angiosarcoma. Although we were unable to perform analyses for local recurrence, we believe that adjuvant radiation will help to control local recurrence and further omit the need of mastectomy.

The CART analysis is an explorative, non-parametric approach, which segregates patients into groups with similar clinical features and survival. Although there are other prognostic methodologies, CART is particularly clinically useful by combinations of clinical characteristics, which can be interpreted as simple rules^21^. It gives a different view of relevant covariate structure of the data, and provides clues to interactions of variables. We selected four-year survival as the cutoff because a longer cutoff time may mask interactions among high-risk factors (e.g. large tumor size, high grade, old age, and metastasis) due to a short survival time associated with those factors. Four variables were selected by the decision tree analysis, which divided PBS patients into nine different risk groups. While tumor size was the most important determinant of long-term survival, age and tumor grade outweigh other TNM staging variables in risk stratifications. Despite different treatment modalities used, the natural course of PBS is largely dictated by patient characteristics and intrinsic tumor biology. The risk stratification model herein proposed is complementary to AJCC 7^th^ staging system in assessing survival outcomes. As an example, patients with stage IV PBS seem to have different 4-year death risk depending on other risk factors, instead of having the same prognosis. The absence of M as a splitter in 50–74 year old patients with T1 tumor size is due to an extremely low number of distant metastasis in this group, which did not meet the pre-specified minimum node size.

Our study is subject to the limitations of SEER database, including the quality of data collection and the number of variables collected. However, SEER registrars are vigorously trained to perform standard and high-quality data collection. Our study is also limited by a retrospective cohort data analysis, instead of a prospective randomized trial. However, the rarity of PBS precludes any attempt of such trials. Additionally, the study patient population was diagnosed from 1973 to 2012. There is significant advancement in medical knowledge and techniques in last 40 years, which has altered the natural course of many tumor types. However, treatment of PBS today is not substantially different from before. Finally, SEER database does not report chemotherapy and tumor recurrence information. We were unable to evaluate impact of chemotherapy on survival outcomes and compare local recurrence rate by different treatment modalities.

In this large population-based cohort study, we demonstrated clinicopathological characteristics of PBS in the US population and validated STS staging system in PBS. We suggested to perform BCS if R0 resection can be achieved and add radiation if tumor size is over 5 cm. If mastectomy is performed, radiation should still be considered for high-risk patients. Further studies are needed to explain the underlying reasons of our findings.

## Materials and Methods

### Data source

Data were obtained from the Surveillance, Epidemiology, and End Results Program (SEER) using SEER 18 dataset of November 2014. Breast sarcoma was identified by ICD-O-3 codes. Variables included patient, pathological and treatment-related characteristics. Of note, the SEER database has not been coded by French Federation of Cancer Centers Sarcoma Group (FNCLCC) grade, and therefore we re-assigned tumor grade by tumor differentiation score using WHO classification of STS.

### Study population

We included women with only one PBS and women with breast sarcoma as the first identified malignancy if there were multiple primary cancers in their lifetime. This is because the etiology and natural history of secondary breast sarcoma may differ significantly from PBS and it is difficult to differentiate primary development from therapy-related breast sarcoma if patients ever received radiotherapy or surgery.

### Statistical analysis

Clinical outcomes of overall survival (OS) and cancer-specific survival (CSS) were used for assessment. Univariable Cox regression analysis was performed to calculate crude hazard ratio (HR) and 95% confidence interval (CI) for death risk, and screen for confounding factors. Multivariable Cox regression was used to assess pathological factors and treatment methods on survival while controlling confounding factors, excluding patients with missing values of related variables. Kaplan-Meier curve was used for cumulative probabilities. Statistical analysis was performed using SAS 9.1 software (SAS Inc, Chicago, IL). A P value of 0.05 or less was considered statistically significant.

We then used a classification and regression tree (CART) analysis to screen for variables useful in patient risk stratification using SPSS software (SPSS Inc, New York). The CART method is an empirical, statistical technique based on recursive partitioning analysis. Unlike multivariable logistic regression, it is well suited to the generation of clinical decision rules. It involves the segregation of different values of classification variables through a decision tree composed of progressive binary splits. Specifically, the recursive procedure starts at the parent node and produces child splits, which in turn become parent node. This process continues until the terminal nodes have no subsequent statistically significant splits or the terminal nodes reach a pre-specified minimum size (n ≥ 5). The optimal tree is determined based on the results of repeated 10-fold cross-validation. In 10-fold cross-validation, the data is first partitioned into complementary subsets called folds. The model is then built on 9 folds and the remaining fold is used as a test-set. This analysis is repeated 10 times, where each of the folds is used as the test-set once. Finally, the estimate of predictive accuracy is calculated from the average performance of the 10 models on their associated test-sets. As a result, CART analysis produces decision trees that are simple to interpret and may be applied at the bedside.

Finally, a second analysis was conducted in which multiple imputation methods were used to fill in missing data for patients who had missing values for 1 or more variables that were included in Cox regression analysis. Five datasets containing observed and imputed data were constructed. Additional analysis was performed independently in each imputated dataset, and a pooled result was combined from parameters from each dataset using PROC MIANALYZE in the SAS statistical software program.

## Additional Information

**How to cite this article**: Yin, M. *et al*. Primary female breast sarcoma: clinicopathological features, treatment and prognosis. *Sci. Rep.* **6**, 31497; doi: 10.1038/srep31497 (2016).

## Supplementary Material

Supplementary Information

## Figures and Tables

**Table 1:** Patient characteristics and survival associations.

Parameters	No. (%)	MST (m)^a^	HR	OS	*P*	HR	CSS	*P*
				95% CI			95% CI	
Age (years)								
≤45	207 (26.4)	463	1			1		
45–75	412 (52.5)	156	1.55	1.18–2.04	0.002	1.16	0.84–1.58	0.37
≥75	166 (21.2)	33	3.87	2.88–5.21	<0.001	1.97	1.37–2.82	<0.001
Race								
Black	89 (11.3)	107	1			1		
White	615 (78.3)	93	1.17	0.84–1.62	0.35	1.72	1.09–2.72	0.02
Asian and Pacific Islander	70 (8.9)	NA^b^	0.73	0.44–1.22	0.24	0.98	0.50–1.94	0.95
Unknown	11 (1.4)	NA^b^		Not Calculated				
Histology								
Angiosarcoma	252 (32.1)	81	1			1		
MFH^c^	62 (7.9)	102	1.06	0.73–1.54	0.75	0.87	0.53–1.42	0.57
Fibrosarcoma	63 (8.0)	247	0.69	0.46–1.05	0.08	0.56	0.32–0.98	0.04
Liposarcoma	47 (6.0)	216	0.54	0.32–0.93	0.03	0.42	0.20–0.86	0.02
Leiomyosarcoma	54 (6.9)	135	1.01	0.64–1.59	0.96	1.01	0.59–1.72	0.98
Spindle cell sarcoma	72 (9.2)	64	1.24	0.86–1.79	0.25	1.13	0.72–1.77	0.59
Osteosarcoma	41 (5.2)	36	1.56	1.00–2.43	0.05	1.41	0.82–2.45	0.22
Sarcoma, NOS	151 (19.2)	97	1.15	0.87–1.52	0.34	0.97	0.68–1.39	0.88
Miscellaneous	43 (5.5)	108	1.25	0.79–1.97	0.34	1.21	0.64–1.97	0.69
Site								
Outer quandrant	213 (27.1)	161	1			1		
Inner Quandrant	95 (12.1)	225	0.86	0.59–1.24	0.40	0.84	0.51–1.37	0.49
Central	40 (5.1)	92	1.11	0.68–1.82	0.67	1.31	0.71–2.45	0.39
Overlap^d^	200 (25.5)	74	1.31	1.00–1.71	0.05	1.5	1.06–2.12	0.02
Entire^e^	226 (28.8)	59	1.47	1.13–1.92	0.004	1.66	1.18–2.33	0.003
Nipple	6 (0.8)	328		Not Calculated				
Unknown	5 (0.6)	107		Not Calculated				
Tumor spread								
Local	569 (72.5)	180	1			1		
Regional	119 (15.2)	29	2.1	1.62–2.71	<0.001	2.59	1.89–3.54	<0.001
Distant	58 (7.4)	8	7.51	5.46–10.32	<0.001	10.17	7.15–14.46	<0.001
Unknown	39 (4.9)	97	1.13	0.69–1.86	0.62	1.07	0.54–2.10	0.85
Surgery								
No	41 (5.3)	34	1			1		
Yes	735 (94.7)	124	0.41	0.27–0.61	<0.001	0.39	0.24–0.64	<0.001
Radiation								
No	545 (70.1)	116	1			1		
Yes	232 (29.9)	108	0.98	0.79–1.23	0.89	1.24	0.95–1.63	0.11

^a^Median survival time by months.

^b^50% death not reached.

^c^Fibrous histiocytoma, malignant.

^d^More than one quandrant but less than half breast size.

^e^Entire breast: ¾ or more of breast involved with tumor, or multiple tumors in different subsites.

**Table 2:** Associations of T, N, M, G prognostic factors with OS.

	No. (%)	MST (m)	Crude HR	95% CI	*P*	Adjusted HR†	95% CI	*P*
T (cm)								
≤ 2	129 (20.5)	197	1			1		
2 to 5	236 (37.6)	133	1.64	1.14–2.37	0.008	1.82	1.24–2.68	0.002
5 to 10	176 (28.1)	37	3.06	2.12–4.42	<0.001	3.48	2.35–5.16	<0.001
>10	86 (13.7)	23	4.03	2.67–6.08	<0.001	4.97	3.18–7.78	<0.001
N								
N0	645 (97.1)	107	1			1		
N+	19 (2.9)	21	2.01	1.15–3.52	0.01	2.60	1.47–4.60	0.001
M								
M0	688 (92.2)	143	1			1		
M1	58 (7.8)	8	6.41	4.69–8.76	<0.001	7.98	5.42–11.75	<0.001
G								
G1	44 (5.6)	340	1			1		
G2	282 (35.9)	221	1.62	0.89–2.93	0.11	1.11	0.53–2.32	0.79
G3	324 (41.3)	42	3.19	1.78–5.73	<0.001	2.57	1.26–5.25	0.009
Gx	135 (17.2)	87	2.79	1.52–5.11	0.001	2.55	1.22–5.35	0.01

^†^Adjusted by age of diagnosis, histology, tumor size, node status, distant metastasis, tumor grade, and surgery.

Patients with missing values of related variables were not included in multivariable analyses.

**Table 3:** Comparison of mastectomy versus BCS by overall survival.

	Crude HR	95% CI	*P*	Adjusted HR†	95% CI	*P*
All M0 patients	1.96	1.48–2.61	<0.001	1.80	1.31–2.47	<0.001
Tumor size						
≤5	1.52	1.05–2.20	0.03	1.52	1.03–2.25	0.04
>5	2.17	1.19–3.95	0.01	2.12	1.13–3.96	0.02
Radiation						
Yes	1.93	1.12–3.35	0.02	1.92	1.03–3.57	0.04
No	1.96	1.40–2.74	<0.001	1.76	1.22–2.55	0.003
Grade						
G1/G2	2.23	1.32–3.74	0.003	2.29	1.23–4.25	0.009
G3/Gx	1.66	1.18–2.33	0.004	1.50	1.03–2.18	0.04
Histology						
Angio	2.54	1.35–4.79	0.004	2.24	1.00–4.98	0.049
Non–angio	1.79	1.30–2.48	<0.001	1.55	1.08–2.21	0.02

^†^Adjusted by age, histology, tumor size, node status, tumor grade, and radiation history.

Patients with missing values of related variables were not included in multivariable analyses.
