# Supplementary material for: Primary female breast sarcoma: clinicopathological features, treatment and prognosis
Source: Sci Rep. 2016 Aug 11;6:31497. doi: 10.1038/srep31497 (PMC4980597; doi:10.1038/srep31497)
Supplement: Supplementary Information [file srep31497-s1.doc]

**Primary female breast sarcoma: clinicopathological features, treatment and prognosis**

Ming Yin^1^, Heath B. Mackley^2^, Joseph J. Drabick^1^& Harold A. Harvey^1^

^1^Division of Hematology and Oncology; ^2^Division of Radiation Oncology, Penn State Hershey Cancer Institute, Hershey, USA.

| **Supplementary Table 1. Associations of T, N, M, G prognostic factors with CSS** | | | | | | | |
| --- | --- | --- | --- | --- | --- | --- | --- |
|  | **No. (%)** | **Crude HR** | **95% CI** | ***P*** | **Adjusted**  **HR**^†^ | **95% CI** | ***P*** |
| T (cm) |  |  |  |  |  |  |  |
| ≤2 | 129 (20.5) | 1 |  |  | 1 |  |  |
| 2 to 5 | 236 (37.6) | 2.51 | 1.43–4.41 | .001 | 2.64 | 1.46–4.78 | .001 |
| 5 to 10 | 176 (28.1) | 5.73 | 3.29–9.95 | < .001 | 5.85 | 3.18–10.77 | < .001 |
| >10 | 86 (13.7) | 7.33 | 4.06–13.21 | < .001 | 6.59 | 2.95–14.73 | < .001 |
| N |  |  |  |  |  |  |  |
| N0 | 645 (97.1) | 1 |  |  | 1 |  |  |
| N+ | 19 (2.9) | 2.62 | 1.46–4.70 | .001 | 3.31 | 1.81–6.02 | < .001 |
| M |  |  |  |  |  |  |  |
| M0 | 688 (92.2) | 1 |  |  | 1 |  |  |
| M1 | 58 (7.8) | 8.1 | 5.77–11.38 | < .001 | 8.43 | 5.51–12.91 | < .001 |
| G |  |  |  |  |  |  |  |
| G1 | 44 (5.6) | 1 |  |  | 1 |  |  |
| G2 | 282 (35.9) | 2.21 | 0.89–5.48 | .09 | 1.24 | 0.44–3.49 | .69 |
| G3 | 324 (41.3) | 4.98 | 2.04–12.17 | < .001 | 3.61 | 1.32–9.82 | .02 |
| Gx | 135 (17.2) | 3.40 | 1.35–8.60 | .001 | 2.63 | 0.93–7.49 | .07 |
| ^†^Adjusted by age of diagnosis, race, histology, tumor size, node status, distant metastasis, tumor grade, and surgery. | | | | | | | |

| **Supplementary Table 2. Associations of treatment modalities with CSS** | | | | | | |
| --- | --- | --- | --- | --- | --- | --- |
|  | **Crude HR** | **95% CI** | ***P*** | **Adjusted HR**^†^ | **95% CI** | ***P*** |
| **M0** |  |  |  |  |  |  |
| T ≤5 cm |  |  |  |  |  |  |
| surgery | 1 |  |  | 1 |  |  |
| surgery+radiation | 1.32 | 0.78–2.24 | .30 | 1.29 | 0.75–2.22 | .36 |
| T >5 cm |  |  |  |  |  |  |
| surgery | 1 |  |  | 1 |  |  |
| surgery+radiation | 0.63 | 0.41–0.97 | .04 | 0.59 | 0.38–0.92 | .02 |
| **M1** |  |  |  |  |  |  |
| surgery (±chemo) | 1 |  |  | 1 |  |  |
| surgery+radiation (±chemo) | 0.64 | 0.31–1.33 | .23 | 0.60 | 0.24–1.53 | .29 |
| chemo or supportive care | 1.24 | 0.55–2.80 | .60 | 0.90 | 0.29–2.87 | .87 |
| **Surgery(M0)** |  |  |  |  |  |  |
| BCS | 1 |  |  | 1 |  |  |
| Mastectomy | 3.10 | 2.04–4.72 | < .001 | 2.57 | 1.63–4.04 | < .001 |
| ^†^Adjusted by age of diagnosis, race, histology, tumor size, node status, tumor grade and radiation history. | | | | | | |

| **Supplementary Table 3. Associations of treatment modalities with survival in imputated data (pooled)** | | | | | | |
| --- | --- | --- | --- | --- | --- | --- |
|  | **Adjusted HR**^†^ | **OS**  **95% CI** | ***P*** | **Adjusted HR^‡^** | **CSS**  **95% CI** | ***P*** |
| **M0** |  |  |  |  |  |  |
| T ≤5 cm |  |  |  |  |  |  |
| surgery | 1 |  |  | 1 |  |  |
| surgery+radiation | 1.15 | 0.76–1.75 | .51 | 1.33 | 0.77–2.29 | .31 |
| T >5 cm |  |  |  |  |  |  |
| surgery | 1 |  |  | 1 |  |  |
| surgery+radiation | 0.59 | 0.40–0.86 | .006 | 0.57 | 0.37–0.88 | .01 |
| **Surgery(M0)** |  |  |  |  |  |  |
| BCS | 1 |  |  | 1 |  |  |
| Mastectomy | 1.65 | 1.25–2.21 | < .001 | 2.36 | 1.55–3.59 | < .001 |
| ^†^Adjusted by age, histology, tumor size, node status, tumor grade, and radiation history  **^‡^**Adjusted by age, race, histology, tumor size, node status, tumor grade and radiation history. | | | | | | |
